# Editorial: Therapeutic potential of propolis—from *in vitro* studies to clinical trials

**DOI:** 10.3389/fphar.2023.1192045

**Published:** 2023-05-25

**Authors:** Otilia Bobiş, Andresa Aparecida Berretta, Miguel Vilas-Boas, David De Jong

**Affiliations:** ^1^ Faculty of Animal Science and Biotechnology, University of Agricultural Sciences and Veterinary Medicine Cluj-Napoca, Cluj-Napoca, Romania; ^2^ Research Development and Innovation Department, Apis Flora Indl Coml Ltd., Ribeirão Preto, Brazil; ^3^ Centro de Investigação de Montanha (CIMO), Instituto Politécnico de Bragança, Bragança, Portugal; ^4^ Ribeirão Preto Medical School, University of São Paulo (FMRP/USP), Ribeirão Preto, Brazil

**Keywords:** bioactive component, propolis, properties, therapeutic potential, editorial

Propolis is a natural product made by bees from bioactive plant exudates and/or resins, which they employ to protect their colony health and integrity (Ghisalberti, 1979; Simone-Finstrom and Spivak, 2010). Taking their cue from the bees, man has been harvesting and using this product for thousands of years, across many civilizations, stimulated by empirical knowledge concerning its efficacy as a medically useful product (Berretta et al., 2020). More recently, the development of analytical methods and modern extraction processes has led to numerous studies regarding the chemical composition and diversity of propolis, and to a better understanding of its therapeutic properties (Bankova et al., 2019). There are numerous scientific publications showing the antimicrobial, antifungal, antiviral, anti-inflammatory, immunoregulatory, and other biological properties of propolis, especially *in vitro* and animal models (Machado et al., 2012; Berretta et al., 2013; Hori et al., 2013; Salatino, 2022). Based on observations from traditional medicine uses of propolis, well-conducted pre-clinical and clinical studies have investigated the therapeutic benefits of propolis for treating a multitude of diseases (Silveira et al., 2019; Berretta et al., 2020; Diniz et al., 2020; Gonçalves et al., 2021; Salatino, 2022). However, routine use of propolis as a health aid requires special care and attention due to the difficulty of guaranteeing a uniform chemical composition, considering that it varies according to plant sources and regions where it is produced (Bankova, 2005; Berretta et al., 2012; Salatino, 2022). Other factors that can affect its properties include the extraction solvents and processes used to produce propolis extracts (Galeotti et al., 2018; Bankova et al., 2021; Suran et al., 2021); these are aspects that potentially also impact their safety and efficacy.

Along this line, the main objective of this Research Topic in Frontiers in Pharmacology was to increase our understanding of how propolis will function in clinical settings, focusing on recent progress in propolis research, to provide an overview of the state of the art in this field. There is considerable evidence that propolis can be therapeutically beneficial, and *in vitro*, *in vivo*, and clinical studies motivated by such evidence have frequently confirmed its utility (Berretta et al., 2020; Salatino, 2022). The continuous use of synthetic medications has resulted in pathogenic bacteria and viruses becoming resistant to these molecules (Salatino, 2022). Consequently, finding alternative options for treatment has become a priority. The various bee products have been intensely studied; among these propolis and its components, with their known properties, have become the principal focus for health research (Salatino, 2022). To help fully understand the potential role of propolis as a health product, pharmacological studies should be encouraged in order to objectively examine the safety and therapeutic benefits of propolis for various diseases and pathologies.

A review paper regarding the suitability of propolis as a bioactive component of biomaterials was included in this Research Topic (Lesmana et al.). This is an innovative approach, since propolis is known mainly as an ingredient in medicinal formulations. This review article analyzes the potential application of propolis as a biomaterial component that can be mixed with other natural or synthetic materials. In the case of orthopedic/dentistry-related materials, two important aspects need to be taken into consideration: osseointegration and protection against infection. Due to the immunomodulatory and anti-inflammatory properties of propolis, it can be considered a useful candidate as a component in biomaterials used in dentistry, including titanium-based biomaterials. Another field in which biomaterials must have certain specific properties is orthopedic applications that use hydroxyapatite/calcium phosphate-based biomaterials. Skin dressing biomaterials used in wound management are important challenges for medicine because mortality from chronic wounds is comparable to that of cancer. The inclusion of propolis in polyvinyl alcohol fiber mats demonstrated that its bioactive compounds were released to the wound. Their healing effects were improved by incorporating silver nanoparticles and propolis into the fiber mats.

Two original research articles (Gonçalves et al.; Rebouças-Silva et al.) were also published in this Research Topic, describing several bioactive properties of propolis (leishmanicidal, immunomodulatory, and cardioprotective). Gonçalves et al. investigated the effects of standardized green propolis extract on heart outcomes of Parkinson rats, through PET imaging to assess cardiac 2-Deoxy-2-18F-fluoro-d-glucose uptake, and by metabolomics to examine the metabolic response to dietary propolis supplementation. The observations include evidence that Brazilian standardized green propolis extract increases cardiac glucose metabolism in propolis-fed rats and showed the relevant metabolic pathways involved in the underlying alterations in metabolite abundance. Propolis supplementation is suggested as a potential treatment to minimize non-motor manifestations of Parkinson’s disease.

Leishmaniasis is part of a group of vector-borne tropical diseases with serious therapeutic limitations. In the study by Rebouças-Silva et al., Brazilian propolis as a standardized product (EPP-AF®) alone and incorporated into a gel formulation was evaluated as an anti-leishmanicidal agent and as an immunomodulator. *In vitro* studies showed that EPP-AF® reduced macrophage infection and modulated the production of various inflammatory biomarkers. *In vivo* analysis demonstrated the effectiveness of a gel formulation containing EPP-AF® for reducing lesion size in infected mice (cutaneous Leishmaniasis).

A clinical trial was also published (Fernandez et al.), in which Brazilian organic propolis extract was used for the treatment of patients after radiotherapy, applied following surgery of head and neck cancer. The use of radiotherapy procedures in oral and oropharyngeal cancer patients can induce acute and chronic toxicities, including oral mucositis, dysphagia, dysgeusia, and oral candidiasis. Finding natural remedies as complementary therapeutics after radiotherapy could help reduce the impact of these side effects on the patients. Organic Brazilian propolis has shown considerable antioxidant, anti-inflammatory and antifungal properties and was used as a complementary therapeutic option for radiotherapy-induced toxicity. Randomized patients received?? an aqueous suspension of propolis or a placebo. Propolis lowered the mean score of toxicity after radiotherapy, and reduced patient pain. Oral candidiasis was also reduced. In conclusion, the topical use of aqueous propolis extract was useful as a complementary option for the prevention and treatment of toxicities resulting from radiotherapy of head and neck cancer patients.

Overall, the published articles in this Research Topic covered various novel and relevant aspects of the therapeutic potential of propolis, portraying new findings that will help strengthen the development of medicinal applications of propolis and its inclusion in clinical practice. We hope that this series of publications will stimulate further studies to improve knowledge in this challenging field.
